# Prescribing antibiotics to ‘at-risk’ children with influenza-like illness in primary care: qualitative study

**DOI:** 10.1136/bmjopen-2016-011497

**Published:** 2016-06-10

**Authors:** Helen F Ashdown, Ulla Räisänen, Kay Wang, Sue Ziebland, Anthony Harnden, for the ARCHIE investigators*

**Affiliations:** Nuffield Department of Primary Care Health Sciences, University of Oxford, Oxford, UK

**Keywords:** PRIMARY CARE, QUALITATIVE RESEARCH

## Abstract

**Objectives:**

National Institute for Health and Care Excellence guidelines recommend immediate antibiotic treatment of respiratory tract infections in ‘at-risk’ individuals with comorbidities. Observational evidence suggests that influenza particularly predisposes children to bacterial complications. This study investigates general practitioners’ (GPs’) accounts of factors influencing their decision-making about antibiotic prescribing in the management of at-risk children with influenza-like illness (ILI).

**Design:**

Qualitative interview study using a maximum variation sample with thematic analysis through constant comparison.

**Setting:**

Semistructured telephone interviews with UK GPs using a case vignette of a child with comorbidities presenting with ILI.

**Participants:**

There were 41 GPs (41.5% men; 40 from England, 1 from Northern Ireland) with a range of characteristics including length of time in practice, paediatrics experience, practice setting and deprivation.

**Results:**

There was considerable uncertainty and variation in the way GPs responded to the case and difference of opinion about how long-term comorbidities should affect their antibiotic prescribing pattern. Factors influencing their decision included the child's case history and clinical examination; the GP's view of the parent's ability to self-manage; the GP's own confidence and experiences of managing sick children and assessment of individual versus abstract risk. GPs rarely mentioned potential influenza infection or asked about immunisation status. All said that they would want to see the child; views about delayed prescribing varied in relation to local health service provision including options for follow-up and paediatric services.

**Conclusions:**

The study demonstrates diagnostic uncertainty and wide variation in GP decision-making about prescribing antibiotics to children with comorbidity. Future guidelines might encourage consideration of a specific diagnosis such as influenza, and risk assessment tools could be developed to allow clinicians to quantify the levels of risk associated with different types of comorbidity. However, the wide range of clinical and non-clinical factors involved in decision-making during these consultations should also be considered in future guidelines.

**Strengths and limitations of this study**

- This qualitative study provides an in-depth assessment of general practitioners’ (GPs’) decision-making processes when faced with ‘at-risk’ children with acute respiratory illness, which has not previously been studied.
- The use of a case vignette to simulate a consultation, with information provided in a structured stepwise way, enabled GPs to discuss their decision-making process as if in real time.
- We sought to obtain a maximum variation sample based on criteria which might affect the antibiotic prescribing decision, such as level of experience in general practice and paediatrics; however, this did not seem to impact eventual decision.
- Using a GP to conduct the interviews, and interviews taking place during a winter of low circulating influenza, may have affected the way GPs handled the case and communicated their opinions.

## Introduction

Children with cough and fever present commonly to primary care general practitioner (GP) services in the UK, particularly in the winter months when there are higher levels of circulating respiratory tract viruses. Approximately a third of these presentations are due to influenza infection.^1^ Each year there are an estimated 490 000 GP consultations owing to seasonal influenza in children aged 14 years or younger.^2^ Testing for influenza infection is not routine in UK general practice, and the term influenza-like illness (ILI) can be used to mean a clinical, rather than confirmed microbiological, diagnosis and includes children with respiratory tract infections (RTIs) caused by other respiratory viruses. ILI can be defined as a fever ≥38°C and cough, with onset in the last 10 days.^3^

For most children, ILI is a mild and relatively short viral illness, but some children can become more unwell or develop secondary bacterial infections such as pneumonia or otitis media. This is more likely to occur in children with pre-existing comorbidities (‘at-risk’ children).^4^ Antibiotics are usually prescribed for children with influenza who already have a bacterial infection (such as pneumonia); however, antibiotics are not generally given to healthy children with ILI who are relatively well, when National Institute for Health and Care Excellence (NICE) guidance for management of RTIs recommends no antibiotics or delayed antibiotics, or an immediate antibiotic if the patient is at high risk of serious complications because of pre-existing comorbidity (which includes patients with significant heart, lung, renal, liver or neuromuscular disease, immunosuppression, cystic fibrosis and young children who were born prematurely).^5^ However, an immediate antibiotic may not be appropriate and is unlikely to be given for an at-risk child who has a simple cold. Observational data suggest that influenza may be associated with greater risk of bacterial infections than other respiratory viruses and early antibiotic treatment of these bacterial infections may improve clinical prognosis.^1^

GPs’ antibiotic prescribing decisions are known to be influenced by diagnostic uncertainty, their own experience and fear of conflict with patients or parents;^6^ ^7^ however, no previous studies have focused on at-risk children. We aimed to investigate what factors influence GPs’ decisions in the management of at-risk children with ILI, particularly in relation to antibiotic prescribing decision.

## Methods

### Setting and recruitment

We aimed to conduct ∼40 interviews with practising GPs in the UK, as we estimated from previous studies using similar methods^8^ that this would be an appropriate number to obtain data saturation. This study was conducted as part of the early use of antibiotics for At-Risk CHildren with InfluEnza in primary care (ARCHIE) programme. We invited GPs from four areas of England (Thames Valley, Bristol, Southampton and Liverpool) in which we subsequently planned to recruit for a randomised control trial. We obtained deprivation and prescribing information from data publicly available via the NHS Information Centre in October 2012^9^ ^10^ and aimed for a maximum variation sample including men and women, a range of lengths of time in general practice and local factors (antibiotic prescribing level and practice population deprivation). GPs were invited to take part in a telephone interview with the first author (who is a GP). The response rate was low (5.8%), so recruitment was extended via local Primary Care Trust (PCT) lists, the RCGP members’ email bulletin, social media groups and primary care research networks. Participants were selected from those who volunteered to obtain a maximum variation sample in terms of the above characteristics. Informed written consent was taken by post. No reimbursement was offered for GP time, although GPs received a certification of participation.

### Data collection

Participants completed a brief questionnaire (sex, length of time working as a GP, special interest in paediatrics, amount of out-of-hours work and whether they had their own children) and practice area (rurality, whether a training practice and whether practice nurses see children with ILI).

Interviews took place between March 2013 and March 2014 and were conducted using telephone or Skype (one was conducted face to face), lasting for ∼20 min (in order to fit in with a busy GP's schedule), conducted by one author (HFA), who is a woman, was a GP ST3/ST4 academic registrar at the time of the interviews and had received formal training in qualitative interview techniques. With the exception of one GP included in the study who was a former colleague, HFA had no prior relationship with the GPs in the study but corresponded by email with participants to pass on the clinical vignette and set up the interview time. Participants were aware that they were speaking to a GP registrar with a research interest in child health and infection. No other individuals were present during the interviews, as far as we were aware.

Interviews were semistructured, and a case vignette was used to focus the discussion: Lily, aged 2, with a medical history of prematurity, atrial septal defect (ASD) and hemiplegia presenting with an acute respiratory illness. Case vignettes are commonly used in UK primary care education and were used previously in qualitative studies.^8^ GPs were emailed the first part of a case vignette before the interview (see box 1) with the child's background and then provided with further information on history and examination findings throughout the interview, in order to mimic how information might be provided in a real-life consultation setting while being flexible to the ways in which different GPs might gather information. We chose this method to help GPs imagine how they might handle such a scenario, and more easily be able to discuss their decision-making process as if in real-time, and deliberately avoided leading the discussion to certain topic areas, rather letting the participant progress through the consultation and discuss their decision-making, for example not raising the possibility of influenza infection or immunisation status until later in the interview, and withholding examination findings in order to discuss how certain potential findings might sway their decision. The case vignette was written to be realistic but to incorporate several different comorbidities, albeit none severe in their own right, making the case less straightforward and to provoke discussion around the importance of different components. GPs were advised not to do any special preparation or revision prior to the interview. The interview topic guide was developed and reviewed by the whole research team, was piloted with two GPs not included in the study and revised in light of feedback.

Box 1 **Case vignette**

**Background information** *(provided before interview)*

You see Lily who is 2½ years old as an urgent extra at the end of a Friday evening surgery. You haven't met the family before but a brief flick of the notes shows that Lily was born prematurely at 32 weeks and spent a week in the special care baby unit for intravenous antibiotics. She was diagnosed with an atrial septal defect, which has been asymptomatic but she continues to be seen periodically at the hospital for serial echos for this. She also has a hemiplegia under follow-up with community paediatrics. Despite this, she's been generally well and she isn't a frequent attender at the surgery.

**Presenting history** *(provided at start of interview)*

Mum has brought her in urgently because she's concerned about Lily. She wouldn't normally bother the doctor but this ‘seems to be more than just a cold’. She's had a runny nose, temperature and cough for the last 2 days but just this afternoon she's seemed more unwell. She can't keep her temperature down with Calpol* and she's become concerned that she seems to be struggling more with her breathing and has vomited once. She's keen to have her checked out and to see if she needs antibiotics.

**Further assessment and examination findings** *(provided during interview after initial discussion; items underlined were provided to all GPs, other items were given only if specifically asked)*

- Off her food for the last day but managing fluids and is passing urine normally;
- Temperatures measured at home up to 39°C;
- Older sister who has just started school has had a cold but has not been this ill with it;
- No rash;
- Immunisations up-to-date including influenza immunisation;
- Lily appears quite grizzly and subdued, preferring to sit on Mum’s knee and not interested in the toys in the corner;
- Appears miserable, but pink and well-perfused;
- Temperature of 38.4°C;
- No signs of respiratory distress;
- Oxygen saturations 97%;
- Chest examination: good air entry with lots of upper airway noise but no crackles;
- Ear, nose and throat examination: heavy green nasal discharge but no obvious focus of infection on examination.

**Interview content**

- Discussion of scenario and thought processes and management of case;
- Extra information to acquire and how this might change management;
- Factors driving decision to prescribe antibiotics or not;
- Importance (or not) of comorbidity;
- Impact of thinking this might be influenza, or in the influenza season;
- Importance of immunisation status;
- Tools or guidelines used in decision-making;
- Advice to parents and safety netting;
- Delayed prescribing;
- Discussion of trial of antibiotics in at-risk children with ILI and how this might change practice.

*Calpol is a widely used term in the UK to describe a popular brand of liquid paracetamol (acetaminophen) sold for children.

Interviews were audio-recorded and professionally transcribed verbatim, then checked for accuracy by the interviewer (HFA). Interview transcripts were reviewed by an experienced qualitative researcher (UR) as recruitment progressed, and the topic guide revised to take account of emerging issues. Transcripts were not returned to participants for comment or correction.

### Analysis

Supervised by the programme's qualitative research lead (SZ), a thematic analysis using constant comparison was used.^11^ The coding scheme was derived from the data. NVivo V.10 software was used for coding, which was conducted by two researchers (HFA and UR) until agreement was reached, and subsequently by one researcher (HFA). The final coding structure was applied systematically to the whole data set (HFA) using NVivo V.10 software. This took place while later interviews were ongoing in order to revise the topic guide in light of identified themes and to establish when data saturation was reached. Codes were then grouped into broader anticipated and emerging issues to develop analytic and conceptual categories. The category of ‘comorbidity’ was then analysed using a mind mapping method to explore patterns in the data as well as deviant cases.^11^ We assessed for any patterns in terms of GP characteristics to explore the variation in GPs’ responses. Further analyses of these data will contribute to the interpretation of an associated trial, within the ARCHIE programme.

## Results

We first discuss the GPs’ awareness of the potential consequences of comorbidity, followed by factors which GPs described as influencing their response to the vignette. A total of 41 interviews were conducted between March 2013 and March 2014; 40 from across England and 1 in Northern Ireland, including one GP trainee. Table 1 shows participant characteristics. Some GPs commented that this was ‘a very common scenario’ and one suggested that this was ‘a really good scenario for discussing whether GPs are going to prescribe antibiotics or not’ (GP22, man, town practice, 25–29 years as a GP).

**Table 1:** Participant characteristics

	n (%)	
**GP characteristics (n=41)**		
Sex		
Man	17 (41.5)	
Woman	24 (58.5)	
Years in general practice since qualification		
<5	9 (22.0)	
5–9	13 (31.7)	
10–14	4 (9.8)	
15–19	1 (2.4)	
20–24	7 (17.1)	
25–29	2 (4.9)	
≥30	5 (12.2)	
Undertake out-of-hours work		
No	26 (63.4)	
Yes—rarely	3 (7.3)	
Yes—sometimes	2 (4.9)	
Yes—often	10 (24.4)	
Special interest in paediatrics*		
No	27 (65.9)	
Yes	14 (34.1)	
Own children		
No	11 (26.8)	
Yes	30 (73.2)	
**Practice characteristics (n=41)**		
Practice area		
Rural	2 (4.9)	
Small town/rural	7 (17.1)	
Town	17 (41.5)	
Inner-city	15 (36.6)	
Training practice		
No	7 (17.1)	
Yes	31 (75.6)	
Not known/not applicable†	3 (7.3)	
Practice nurses see children with influenza-like illness		
No	20 (48.8)	
Yes	16 (39.0)	
Not known/not applicable†	5 (12.2)	
Practice list size (n=38)†‡	Median (IQR)	7788 (5986)
	Range	2455–38 532
	England average	6845
Per cent of children under 18 registered (n=38)†‡	Median (IQR)	21.1% (3.9%)
	Range	12.7–36.7%
	England average	20.8%
Index of Multiple Deprivation (n=38)†‡§	Median (IQR)	27.5 (20.2)
	Range	4.3–49.4
	England average	21.5

*Details of special interests described included hospital paediatrics experience, the Diploma in Child Health and responsibility for child health surveillance/baby clinics within the practice.

†Data not available for some characteristics as three locum GPs included in the study, or where details not completed.

‡Practice list size, deprivation score and percentage number of children under 18 registered at the practice were recorded using the Public Health England National General Practice Profiles^28^ on the date of recruitment. Average values for England were recorded at the start of the interviews in March 2013.

§Index of Multiple Deprivation provides information on relative levels of deprivation in England and ranges nationally from 2.9 (lowest deprivation) to 68.4 (highest deprivation). Lower Layer Super Output Area level deprivation data are applied proportionally to the Attribution Data Set practice populations.^28^

IQR, inter-quartile range.

There was a large variation in the degree of certainty with which GPs responded to the vignette—some seeing it as quite routine.

You see goodness knows how many we see and by and large most of these are viral presentations… So we would sort of go through that, safety netting, out of hours, in fact we've got a leaflet which we tend to hand out, especially to paediatric under-fives. (GP33, man, small town/rural practice, 5–9 years as a GP)

Although many recognised that this was a more challenging case, ‘a very grey sort of area’, and expressed uneasiness about assessment and management:

I'm feeling uneasy about it because she's not quite a straightforward lively healthy toddler who's got a cold; there's a bit more going on here, or a bit more potentially going on. (GP08, man, inner-city practice, ≥30 years as a GP)

I think it's just hugely difficult… It's a complete nightmare… you sit there in practice and you think, ‘Well, how on earth can you decide whether it's viral or not?’ (GP26, woman, small town/rural practice, ≥30 years as a GP)

### Awareness of the potential consequences of comorbidity

GPs recognised that some comorbidities might be associated with an increased risk of poor outcomes following an RTI, in terms of developing a more severe infection and risk of a secondary infection. Some said they aimed for prevention as well as treatment.

And even though it might not be causing a problem while they're fit and well, if they get a really bad infection then that can cause them some difficulties and they could actually die. (GP27, woman, rural practice, 5–9 years as a GP)

Because they do get unwell more quickly. And that is the experience, they'll go in with a pneumonia or a chest infection and it just started off as a cold. So, you know it does change depending on their past medical history, it can change things quite dramatically. (GP32, woman, inner-city practice, 5–9 years as a GP)

Although GPs suggested that comorbidity would lower their intervention threshold, it was rarely described as an important part of the assessment. GPs varied in terms of which specific comorbidities seemed most important.

#### Atrial septal defect

There was uncertainty about the significance of the ASD and risk of cardiac or respiratory decompensation because this required follow-up yet was asymptomatic. Concerns included whether there would be an increased risk of endocarditis or rheumatic fever, which might require antibiotic prophylaxis.

#### Prematurity

GPs associated prematurity with being ‘more vulnerable’ and with increased susceptibility to significant respiratory infections:

I mean they, they often have had a history of respiratory distress haven't they, sort of in those first few weeks. And they're the kind of babies that tend to get bronchiolitis when they're little. […] I think that they are more susceptible to chest infections and to getting more sickly so, so yeah I would certainly take that into account. (GP04, woman, inner-city practice, 10–14 years as a GP)

#### Hemiplegia

For a few GPs, the hemiplegia was thought to potentially affect mobility, making children harder to assess. If they were less active, they might have difficulty in clearing secretions and therefore develop respiratory infections.

I'd be wondering whether the hemiplegia had a sort of effect on her general mobility… I might be thinking […] is this a child that's not very active and that's going to be a bit more vulnerable to developing a serious respiratory infection…if you see a child for example in a wheelchair, you just wonder about how their respiratory muscles, how sort of fit they are almost. And whether actually their ability to move secretions out of their lungs and you know run around and take lots of exercise is going to make them a bit more vulnerable. I mean that sounds like a bit of sort of, I don't know, pseudo-science really but it, it's kind of borne out of experience as well. (GP29, woman, town practice, 25–29 years as a GP)

#### Other comorbidities

In contrast, comorbidities in this case were seen as ‘slightly soft’ and would not necessarily affect an acute presentation of this nature. Some GPs contrasted other comorbidities that they would regard as more significant, including other chronic neurological problems, Down syndrome, diabetes, cystic fibrosis and metabolic disorders relating to consanguinity.

#### Age

GPs were reassured by the lack of problems since the neonatal period and by Lily's age (two and a half).

If she was 8 weeks old it would probably make a lot more difference… I think at eight weeks they are likely to decompensate much more quickly than they are at age two and a half. They've got less reserve. So I'd probably be more cautious the younger the child. […] And I don't know, I think just a general feeling that I'd be less comfortable treating a child with congenital heart disease who's very young compared to one who's toddler age. (GP05, man, town practice, 10–14 years as a GP)

### Clinical assessment guiding antibiotic prescribing decisions

All GPs said that they would want to further assess the child in person: it was seen as ‘a justified request’ to be seen as an extra on a Friday. In terms of management decision after assessment, different GPs discussed a range of possibilities that could be described as a ‘spectrum of interventions’ (sometimes in combination), increasing from reassurance and safety netting, through arranging a GP review later, delayed antibiotic prescribing, immediate antibiotic prescribing, telephone discussion with paediatrics, to immediate hospital referral. Some GPs wanted to start antibiotics earlier in the course of illness than they would with a healthy child. The only GP who wanted to arrange hospital assessment after having heard all of the clinical information was concerned that the ‘subdued’ description might indicate meningitis, and in the context of an ASD she suggested specialist paediatric assessment.

Some GPs talked about the importance of identifying a bacterial infection, with the assumption that this would definitely require antibiotics or hospital assessment. Only a few GPs brought up potential influenza spontaneously, and most felt that identification of the illness as potential influenza versus ‘A N Other viral thing’ was less important in a non-pandemic setting, with GPs tending to assess and manage as a generic RTI. Likewise, Lily's immunisation status was rarely mentioned spontaneously, and on direct questioning few GPs saw influenza immunisation as particularly relevant to the scenario.

Lily's comorbidity worried some of the GPs who were concerned that they might miss a serious diagnosis. This might increase their likelihood of prescribing antibiotics, which was widely seen as the safer, easier and more ‘risk averse’ course of action. Antibiotic resistance and antibiotic side effects were frequently mentioned, but mainly as an external issue, for example:

It's one of those grey areas where one dreads making a mistake, where you've got conflicting forces, sometimes parental expectation which is, you know in a sense is almost a side issue, but the awareness that inappropriate antibiotics is a major problem but missing one child with a pneumonia who then gets ill or dies is ****ing disaster… […] The easiest thing is to dish them out and not worry about the global issue. (GP08, man, inner-city practice, ≥30 years as a GP)

What all that [discussion of co-morbidities] builds up to is it's a brave GP who just simply says “Look this is an upper respiratory tract infection, it's okay not to do anything extra.” (GP22, man, town practice, 25–29 years as a GP)

#### Lily's case history and examination

Lily's past experience of illness and how she seemed on the day were seen as important. GPs variously said that they would look through Lily's notes and/or ask the parent to check whether there were specific instructions from hospital specialists.

You know perhaps one of the previous letters from the cardiologist might even say something like, you know, please keep a low threshold for giving antibiotics or referring back if she's deteriorating. So I'd want to have a quick scan through hospital letters, see if I can see any little informative nuggets like that. (GP35, man, town practice, 20–24 years as a GP)

GPs wanted to establish Lily's typical trajectory of RTIs, in terms of duration, severity, previous hospital admissions (including any intensive care admissions) and what had helped before, including the previous antibiotic prescribing pattern.

The response to the clinical vignette usually included a structured examination, including vital signs (fever, respiratory rate and heart rate), pulse oximetry (although not always available for children) and examining for respiratory distress or chest crepitations. Abnormalities in these areas would tend to raise concerns of a more serious illness and push the GP towards hospital assessment. However, more important in the decision to prescribe antibiotics was the global assessment of ‘how she is in herself’:

I think I would probably would just play the child in front of me and be more guided by how they were, how worried I was about them at that point rather than their history. (GP19, man, inner-city practice, <5 years as a GP)

As mentioned above, all of the GPs said that they would want to see Lily and some commented that when they did their ‘gut feeling’ would sway their decision about prescribing.

#### Mutual trust and confidence with the parent

GPs often mentioned the need to establish the parental concerns and work out whether this was a ‘sensible’ parent. The number and nature of previous GP attendances for similar illnesses were also considered important, and many commented that parents of children with comorbidity might have different experiences, expectations and ability to manage their child's illness than other parents.

And what I'm picking up now is that Mum thinks she's different to usual. So there's parental concern, in my experience of looking after and supporting the families of babies who've had a difficult time or have been, spent a lot of time on the special care baby unit, is that they are very very expert parents, and so I think I would take the fact that she's an infrequent attender, but has come in significantly concerned that she's really not herself very seriously, I would be quite worried about that […] So often parents who've had a child who's had one episode of pneumonia, that experience will influence how they feel about other coughs and fevers. So I think I would explore all of that with Mum. (GP09, woman, inner-city practice, 10–14 years as a GP)

Others characterised these parents as potentially overanxious and overcautious, which could affect how they handled the case.

I think this is sort of premature babies sometimes get sort of wrapped in cotton wool a little bit, and thought to be a little bit more precious, but it's just like any other child really but they sort of, they carry with them for the first few years that sort of, almost like slightly precious “need protecting”-type approach sometimes. (GP31, man, inner-city practice, 10–14 years as a GP)

If Lily's mother was seen as a ‘sensible parent’, this would probably affect how they incorporated the mother's opinion, and how much responsibility could be expected in monitoring the child for signs of deterioration and returning for further assessment.

I'm kind of, you know, if they're sick they need to be treated. If they're not sick they need to be told to come back. And be given a lot of permission to do that and I'm forever saying “If you're worried, I'm worried,” to patients, to Mums and Dads. To really underline you know, “You're the world's expert,” is the other thing that I'm always forever saying, “You're the world's expert on your child.” (GP21, woman, inner-city practice, 5–9 years as a GP)

Education, culture and language as well as other sources of family support were factors contributing to the assessment of the parent. Some stated that they were assuming that Lily's mother was a ‘coper’ or ‘capable’ from the history described. Continuity was mentioned as an important factor for some GPs: if they had a longstanding and trusting relationship with the family and knew the child's history, they would feel more confident about managing this episode. Others talked about sharing the decision about antibiotic prescribing with the parent if they were uncertain about the best management.

#### GP's confidence and experience

Some GPs acknowledged that particular memorable cases had impacted on their current practice or saw themselves as more or less experienced in assessing children. Some mentioned that they were inexperienced in paediatrics and that this might affect how they handled Lily's case. However, relative experience in paediatrics did not seem to predict eventual management decisions. Only a handful of GPs (particularly those more recently qualified) mentioned using guidelines or other tools to help with decision-making, although on specific questioning about this, many GPs were aware of the Centor criteria for sore throats^5^ and NICE guidelines (many mentioned the traffic light system for assessment of a feverish child,^12^ but none alluded to the guidance on comorbidities in relation to antibiotic prescribing for RTI).^5^

But I am aware that ASDs are one of the things where I think there is still a lower threshold for prescribing antibiotics, but I would feel out of my comfort zone as to know when that was. So I would be, I think because I haven't come across an ASD for years I think I would still probably just ring the paediatricians and put it past them to check that, because I'm not quite clear about what the guidelines are with ASDs so I'd feel more comfortable just ringing them and asking them. (GP41, woman, small town/rural practice, 20–24 years as a GP, no reported specialist expertise in paediatrics)

Generally speaking you know I'm not impressed by the scoring systems, I've done quite a lot of paediatrics, I'm pretty confident about spotting sick children. I'm pretty confident about guiding parents about what to do in case I've actually got it wrong and given false reassurance. So I'm pretty confident with all that sort of stuff. (GP02, man, town practice, ≥30 years as a GP, some specialty training in paediatrics)

GPs self-identified as being high or low antibiotic prescribers, and this could also relate to how recently qualified the GP was.

I think down to my own experience and where I feel I am in my practice compared to my peers, it's quite interesting how I use antibiotics quite a lot less and I wonder if that's because I have actually seen people die in hospital, in my career, from bacterial, you know antibiotic resistance and C.Diff diarrhoea and things like that. […] And I've noticed that talking to my more senior colleagues who are probably 10, 20 years my senior, they've never seen the sort of deaths that I've discussed in hospital. So I think that has a big effect on what I do. (GP07, woman, small town/rural mix practice, 5–9 years as a GP)

In contrast, one GP with paediatrics experience said that ‘we don't see sick children like they used to in general practice” (GP02), and more recently qualified doctors would not have seen serious complications of vaccine-preventable infections.

#### Other factors

The setting of this case on a Friday evening was important and affected prescribing decisions (although some said they were consciously trying to ignore this feature of the vignette).

I think the problem with this is obviously ‘cos it's a Friday evening. A Friday morning and you can still say well see how she goes over the next couple of hours and if there's any problem then bring her back, but a Friday evening if you're not in on the Saturday makes it, that judgement a little bit harder. (GP10, woman, town practice, 20–24 years as a GP)

Friday evening surgery the threshold for prescribing antibiotics and getting rid of the patient that much more quickly is lower. (GP22, man, town practice, 25–29 years as a GP)

Local circumstances and priorities, including out-of-hours services and proximity to Lily's home, and the quality of the out-of-hours service were all cited. One rural GP sometimes gave her own telephone number to parents she knew well for use out-of-hours, owing to distance from other services. Others said that they would prescribe to help reduce pressure on the out-of-hours service over the weekend or, where this facility existed, arrange for a formal review of a child out-of-hours.

### Delayed prescribing

Delayed prescribing was the most divisive issue. Some GPs wanted to give a delayed prescription so that treatment was started earlier if the child did not improve (particularly in the context of an upcoming weekend), whereas others preferred to review the child if there was no improvement or if they were not confident that the parent would return in the event of deterioration if they already had a prescription.

## Discussion

### Principal findings

This is the first study of the impact of comorbidity on GPs’ assessment of children with ILI. There was uncertainty and variation in opinions about whether, and to what extent, long-term comorbidities are associated with increased risk in children presenting with ILI and considerable variation in management among GPs. Actions included watch-and-wait, immediate or delayed antibiotic prescription and hospital referral. Analysis of GPs’ responses to the clinical vignette identified several factors influencing their decisions including the child's history, current appearance, mutual trust and confidence between the GP and the parent, the GP's own confidence and experience and arrangements for weekend care locally. Neither suspicion of potential influenza infection nor immunisation status was described as an important factor affecting assessment or management. Guidelines were rarely mentioned: more important was the GP's global impression of the child, incorporating the above factors to varying degrees. All wanted to see the child. Some participant characteristics, such as previous paediatric experience and rurality, played a role in decision-making but did not predict ultimate management plan and antibiotic prescribing decision.

### Strengths and limitations

We sought a diverse sample of GPs with a range of experiences and practice characteristics. Although we can say nothing about the frequency with which these findings would appear in the wider population, a wide range of views was evident in responses. Our use of a case vignette involving comorbidities and an uncertain degree of risk enabled us to demonstrate the variation in clinical management. Providing case information in a stepwise manner enabled us to replicate the process of a consultation. Recruitment via mailing lists extended geographical diversity but made it more likely that those GPs who responded had particular special interests or experiences, leading to findings that may represent less variation than might be found in the wider GP population.

Very few GPs mentioned influenza as a potential cause of the RTI until specifically prompted, despite the study title including the term ‘influenza-like illness’, and GPs tended not to differentiate between influenza and other viruses in assessment or management. The interviews were conducted in all seasons, including a winter of low influenza incidence in the UK,^13^ and it is possible that influenza was less in the forefront of GPs’ minds.

Knowing they were talking to a GP colleague may have encouraged participants to give a preferred response or collaborate in assumptions about the case vignette, but there was considerable expression of uncertainty and little suggestion that participants were drawing on guidelines in their responses. Interview transcripts were reviewed by a qualitative researcher early in the data collection period to make sure that the interview did not resemble a test.

We did not interview practice nurses, although a significant minority (39.0%) of GPs reported in their preliminary questionnaire that practice nurses did see children with ILI. On further questioning about this, many said that practice nurses would be unlikely to assess a child with significant or complex comorbidities, and a recent qualitative clinician interview study found no differences in practice between practice nurses and GPs.^7^

### Comparison with existing literature

The Department of Health recommends influenza vaccination in specific groups who are considered to be at high risk of serious complications,^14^ and NICE guidance recommends an immediate antibiotic prescription ‘if the patient is at high risk of serious complications because of pre-existing comorbidity. This includes patients with significant heart, lung, renal, liver or neuromuscular disease, immunosuppression, cystic fibrosis and young children who were born prematurely’.^5^ A systematic review and meta-analysis found that strong risk factors for hospital admission due to influenza-related complications included neurological disorders, prematurity, diabetes and age under 2 years, as well as the presence of more than one risk factor, which is similar to the specific comorbidities GPs brought up based on their experience.^15^

Similar to our findings, a systematic review and metaethnography of antibiotic prescribing for RTIs in all ages found that treatment strategies varied between GPs, and this was affected by their previous experience, uncertainty about diagnosis, ease of follow-up and fear of consequences of non-prescribing, as well as perceptions of potential conflict with patients.^6^ Another qualitative systematic review in children found a particular focus on relationship with the parent, particularly perceived pressure to prescribe and consequences for the future doctor–patient relationship, although this was based on clinical need as much as the need to preserve this future relationship.^16^ GP perception of patient expectation is strongly associated with a decision to prescribe antibiotics,^17^ ^18^ and it may be that the construction of Lily's mother as an ‘expert parent’ would lead to assumptions about her antibiotic expectations and changed the dynamic of the consultation (and some GPs commented on this assumption). However, GPs’ tolerance of antibiotic prescribing conflict varies,^19^ and this may account for more of the difference in eventual decision than other GP characteristics. Our finding of individual versus global risk assessment in decision-making was also found in a recent cross-study qualitative analysis^20^ of prescribing behaviour for children with RTIs, which identified antibiotic prescribing as being the safer option to manage clinician uncertainty: an unnecessary antibiotic prescription was perceived as less of a threat to professional standing and the child's health than a missed serious diagnosis. GPs were also more likely to prescribe if parents were not judged to be adequate ‘risk managers’ for the child, similar to our study with the concept of ‘a sensible parent’. Quantitative studies on antibiotic prescribing for respiratory infections in adults have also found considerable variation in antibiotic prescribing, which cannot be explained by variation in clinical presentation.^21^

In terms of clinical assessment, our findings are similar to a study of clinicians’ antibiotic prescribing behaviour for lower RTIs in adults,^22^ where GPs discussed combining different clinical and patient preference factors, giving these different weightings that contributed to a tipping point for prescribing. However, most important for many GPs was the global assessment of how the child appeared in herself, hence the need to see her at the practice. This was also found in a recently published qualitative interview study of clinicians, which described a rapid initial assessment based on pattern recognition, then a more formal deductive process.^7^ Clinicians’ ‘gut feeling’ is well established to be of high diagnostic value in assessing serious infections in children, with high correlation with child's overall response and breathing pattern.^23^ Qualitative work has shown that GPs use gut feelings (of either reassurance or alarm) as a compass in situations of uncertainty;^24^ GPs in our study discussed multiple factors, which might inform their decision-making, but it may be that this gut feeling is the eventual sway to decision-making. Hence the need to see the child rather than deal with the consultation entirely by telephone. The recently published clinician qualitative interview study mentioned above had similar findings in many other areas, including uncertainty over managing intermediate illness severity, difficulty differentiating bacterial from viral infection clinically and GPs’ self-confidence and experience in paediatrics.^7^ Some GPs in our study discussed parental pressure to prescribe, but generally this was seen as less important. This may have been because our study particularly focused on comorbidity, with GPs identifying ‘expert parents’ and their ability to manage uncertainty appropriately. Evidence from studies with parents suggests that parents are not necessarily seeking antibiotics but prefer to defer this decision to the clinician.^16^ ^20^

### Implications for clinicians, policymakers and future research

Our study suggests that, faced with a clinical scenario, GPs expressed wide variation and uncertainty about how to manage at-risk children with acute RTI. Current guidelines leave great scope for interpretation, and lists of comorbidities are not exhaustive, such that clinical judgement balancing multiple factors is required to assess and quantify the levels of risk. Tolerance of uncertainty is a key facet of a skilled GP,^25^ and while some GPs were inclined to defer to parental opinion or (in one case) to hospital referral, others seemed to think that they would be abrogating their clinical responsibility if they reacted to uncertainty in this way. However, this study suggests that this is an area in which further research and additional guidance are needed to help bridge this gap. Particularly, development of risk assessment tools might allow clinicians to quantify the risk associated with different types of comorbidities and the presence of multiple conditions and weigh this against the potential more general risks and benefits of early antibiotic prescribing. This might facilitate more consistent and accurate antibiotic prescribing among healthcare professionals who assess these types of scenarios. Also notable was that GPs did not view influenza differently from other causes of RTI in children, despite the greater risk of bacterial infections.^1^ This may be an area for further research into diagnostic accuracy of clinical features of influenza, potential training and educational interventions and/or assessment of whether there may be a greater role for rapid point-of-care tests for influenza in UK primary care.^26^ Decisions about antibiotic prescribing are particularly important in the context of a pandemic, where hospital capacity may be an issue. At-risk individuals make up a larger proportion of those presenting with ILI than found in the general population,^4^ with over 70 000 consultations estimated to take place for ‘at-risk’ children with ILI,^4^ ^27^ and so this may be a particularly worthwhile group to target for such interventions.

When incorporating any new evidence into practice and producing guidance in this area, it will be important to take into account the factors raised here, and inclusion of non-clinical factors such as parental experience is likely to be particularly important in what will undoubtedly remain a complex decision-making process.
